# In Vitro Pharmacological Characterization of RXFP3 Allosterism: An Example of Probe Dependency

**DOI:** 10.1371/journal.pone.0030792

**Published:** 2012-02-07

**Authors:** Lily Alvarez-Jaimes, Steven W. Sutton, Diane Nepomuceno, S. Timothy Motley, Miroslav Cik, Emily Stocking, James Shoblock, Pascal Bonaventure

**Affiliations:** Janssen Pharmaceutical Companies of Johnson & Johnson, San Diego, California, United States of America; University of Oldenburg, Germany

## Abstract

Recent findings suggest that the relaxin-3 neural network may represent a new ascending arousal pathway able to modulate a range of neural circuits including those affecting circadian rhythm and sleep/wake states, spatial and emotional memory, motivation and reward, the response to stress, and feeding and metabolism. Therefore, the relaxin-3 receptor (RXFP3) is a potential therapeutic target for the treatment of various CNS diseases. Here we describe a novel selective RXFP3 receptor positive allosteric modulator (PAM), 3-[3,5-Bis(trifluoromethyl)phenyl]-1-(3,4-dichlorobenzyl)-1-[2-(5-methoxy-1H-indol-3-yl)ethyl]urea (135PAM1). Calcium mobilization and cAMP accumulation assays in cell lines expressing the cloned human RXFP3 receptor show the compound does not directly activate RXFP3 receptor but increases functional responses to amidated relaxin-3 or R3/I5, a chimera of the INSL5 A chain and the Relaxin-3 B chain. 135PAM1 increases calcium mobilization in the presence of relaxin-3_NH2_ and R3/I5_NH2_ with pEC50 values of 6.54 (6.46 to 6.64) and 6.07 (5.94 to 6.20), respectively. In the cAMP accumulation assay, 135PAM1 inhibits the CRE response to forskolin with a pIC50 of 6.12 (5.98 to 6.27) in the presence of a probe (10 nM) concentration of relaxin-3_NH2_. 135PAM1 does not compete for binding with the orthosteric radioligand, [^125^I] R3I5 (amide), in membranes prepared from cells expressing the cloned human RXFP3 receptor. 135PAM1 is selective for RXFP3 over RXFP4, which also responds to relaxin-3. However, when using the free acid (native) form of relaxin-3 or R3/I5, 135PAM1 doesn't activate RXFP3 indicating that the compound's effect is probe dependent. Thus one can exchange the entire A-chain of the probe peptide while retaining PAM activity, but the state of the probe's c-terminus is crucial to allosteric activity of the PAM. These data demonstrate the existence of an allosteric site for modulation of this GPCR as well as the subtlety of changes in probe molecules that can affect allosteric modulation of RXFP3.

## Introduction

RXFP3, also known as GPCR 135 [1] or the SALPR [2], is the cognate receptor for relaxin-3 (Relaxin-3). Relaxin-3 and related receptors will be referred to by their IUPHAR terminology [3]. RXFP3 is a class A, G_i/o_ linked GPCR with an extensive distribution in the central nervous system (CNS), particularly in areas involved in memory, sensory and emotional processing [4]. The CNS distribution of RXFP3 is conserved in mice and macaques [5], [6]. RXFP4, the INSL5 receptor, is the closest homolog to RXFP3 [7].

Relaxin-3 is a member of the insulin superfamily, which includes insulin, insulin-like growth factor, the relaxin hormone (H1/H2 Relaxin) and insulin-like peptides (INSL-3, 4, 5 and 6). In addition to RXFP3, relaxin-3 is capable of high affinity interaction with RXFP4. However, RXFP4 and its native ligand (INSL5) are thought to be more active in the periphery [7]. RXFP4 is a pseudogene in the rat [8] and does not appear to be expressed in the mouse brain [9]. While Relaxin-3 shares part of its name with H1/H2 Relaxin, it has a distinct distribution and roles compared to the hormonal H1/H2 Relaxin. H1/H2 Relaxin acts through RXFP1 [10] and has important roles in collagen remodeling, impacting diverse physiological processes from pregnancy [11] to asthma [12] and heart failure [13]. Relaxin-3 is highly and focally expressed in the nucleus incertus of the hind brain of the mouse [6], [14], rat [1], and an equivalent area of the macaque [5]. Apart from the Relaxin-3 distribution, behavioral data suggest that Relaxin-3 has a role in stress/anxiety [15], cognition [16] and appetite regulation [17], [18]. The tissue distribution and function of Relaxin-3 and RXFP3 indicate potential therapeutic application of RXFP3 modulators to treat stress/anxiety, cognitive disorders and metabolic diseases [19]. In vivo agonist stimulation of RXFP3 in rodents increases hippocampal theta emissions and improves performance in behavioral cognitive assays [16], [20].

Selective orthosteric, high affinity agonist (R3/I5) and antagonist (R3(Δ23–27)R/I5) peptides have been discovered for RXFP3 [21], [22]. The selective agonist (R3/I5) pairs the INSL-5 A chain with Relaxin-3's B chain, yielding a selective, high affinity, full RXFP3/4 agonist. Residues 23 through 27 of the Relaxin-3 B chain are necessary for agonist activity and removing those resides from R3/I5 creates a competitive antagonist (R3(Δ23–27)R/I5). While these peptide modulators of RXFP3/4 have been useful, they must be administered i.c.v. Small molecule drugs for these receptors have yet to be described in the literature.

GPCR agonists and antagonists can act either orthosterically, at the same site as the native agonist, or allosterically at a separate site on the receptor. Positive allosteric activity can take the form of allosteric agonism (activity in the absence of an orthosteric agonist) or positive allosteric modulation (PAM; requires presence of an orthosteric agonist) [23], [24], [25]. Positive allosteric modulators have several theoretical advantages over direct agonists as therapeutic agents. As PAM compounds modify the efficacy of an orthosteric agonist and lack activity on their own, these compounds are expected to have an effect only in tissues where and when the endogenous agonist is released. The ability of PAM compounds to exhibit biological activity in a regulated manner enhances their safety profile and makes them attractive tools over traditional therapeutic agents [26].

**Figure 1:**
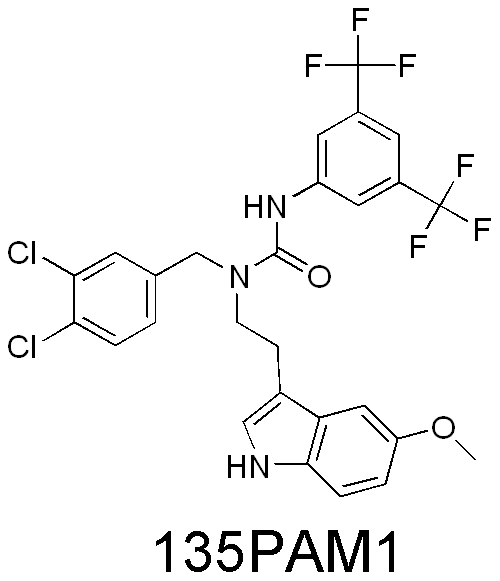
Structure of 135PAM1 (3-[3,5-Bis(trifluoromethyl)phenyl]-1-(3,4-dichlorobenzyl)-1-[2-(5-methoxy-1H-indol-3-yl)ethyl]urea).

In the case of positive allosteric modulation, the allosteric activity is measured in the presence of an orthosteric agonist, referred to as a probe. Examples of PAM compounds with probe-specific activities have been described in the literature [27]. Here we describe a RXFP3 PAM compound (135PAM1, Figure 1) which is a striking example of probe selectivity. In this case, one can exchange the entire A-chain of the probe peptide while retaining PAM activity, but the state of the probe's c-terminus is crucial to allosteric activity of the PAM.

## Materials and Methods

### Materials

All cell culture supplies, Fluo-3 AM calcium dye and Pluronic acid F-127 were obtained from Invitrogen (Carlsbad, CA). Probenecid and forskolin were obtained from Sigma (St. Louis, MO). Chlorophenol red-β-D-galactopyranoside (CPRG) was purchased from Roche Applied Science (Indianapolis, IN). C-terminally amidated Relaxin-3 (relaxin-3_NH2_) and R3/I5 (R3/I5_NH2_) were obtained from Dr. John Wade at the Howard Florey Institute (Melbourne, Australia). The C-terminal free acid forms of Relaxin-3 and R3/I5 were purchased from Peprotech (Rocky Hill, NJ) and Phoenix Pharmaceuticals (Burlingame, CA), respectively. For these studies both the A and B chains of the peptide were amidated (i.e. Relaxin-3_NH2_) or left in the free acid form (i.e. Relaxin-3_OH_). Amidated R3/I5 (R3/I5_NH2_) was radioiodinated with Na^125^I using the chloramine T methodology as previously described [28]. 135PAM1 (3-[3,5-Bis(trifluoromethyl)phenyl]-1-(3,4-dichlorobenzyl)-1-[2-(5-methoxy-1H-indol-3-yl)ethyl]urea) was synthesized and prepared at Johnson & Johnson Pharmaceutical Research and Development, L.L.C.

### Cell Lines

Stable cell lines expressing RXFP3 and RXFP4 were created as previously described [1], [8], [29]. Clonal, stable cell lines were made using the receptor genes of interest, cloned into pCIneo (Promega, Madison, WI). For functional assays measuring inhibition of the cAMP pathway, the receptor constructs were transfected into SK-N-MC cells (ATCC, Manassas, VA) expressing a CRE reporter linked to β-galactosidase expression. Other assays were performed using stable clones of HEK-293 cells (ATCC, Manassas, VA) coexpressing the receptor gene of interest (RXFP3 or RXFP4) and chimeric G_α_ protein G_q_I_5_ [30].

### Ca^2+^ Mobilization Assay

Intracellular Ca^2+^assays were used to assess the effect of 135PAM1 to either increase the response to a probe quantity of an agonist (∼EC20) or to measure changes in an agonist's EC_50_ at various compound doses. In either case, adherent HEK-293 cells expressing G_q_I_5_ were grown to confluency in DMEM media supplemented with 10% fetal bovine serum, 50 iu/ml penicillin, 50 µg/ml streptomycin, 1 mM Na Pyruvate, 10 mM HEPES, and 600 µg/ml G418. On the day before the assay the cells were detached with 10 mM trypsin/EDTA and seeded on black poly-D-lysine-coated 96-well plates (BD Bioscience, San Jose, CA) at a density of 35,000 cells/well, then incubated overnight at 37°C, 5% CO_2_. On the next day, the culture medium was discarded and replaced with a calcium dye solution, containing 4 µM Fluo-3 (AM), 2.5 mM probenecid and 0.04% Pluronic acid F-127 in DM:F12 medium. Cells were incubated in the dye solution for 60 min at room temperature or 30 min at 37°C. Ligand-stimulated Ca^2+^ mobilization was monitored using FLIPR Tetra High Throughput Cellular Screening System (Molecular Devices, Sunnyvale, CA).

An initial assay assessed the effect of 135PAM1 to increase the effect of an EC_20_ dose of Relaxin-3 or R3/I5 (each as the c-terminal acid or amide). In this case, the cells were incubated with a dose-response of 135PAM1 (0 to 20 µM) for 10 min at room temperature, then Relaxin-3 or R3/I5 was added by the FLIPR Tetra (Molecular Devices, Sunnyvale, CA) as Ca^2+^responses were recorded.

Shifts in the dose response curve of the orthosteric agonist were assayed using the HEK-293 cell lines co-expressing G_q_I_5_. In this case, the cells were incubated with 135PAM1 at 0, 0.2, 2 and 20 µM for 10 min at room temperature. The FLIPR Tetra then added full dose-response curves of the orthosteric agonist (H3 relaxin or R3/I5; 0 to 200 nM) at each concentration of 135PAM1 while recording data.

Results of 3 or more triplicate assays were plotted and calculated using GraphPad Prism version 5.03 (GraphPad, La Jolla, CA). The allosteric site K_b_ was calculated from agonist curve shift assays using the Christopoulos/Kenakin equation [23]. This calculation accounts for the limiting character of the shift in the orthosteric agonist's EC_50_ due to allosteric modulation. Results were analyzed together after normalization and are reported with confidence intervals in parenthesis.

### cAMP Reporter Assay

Alterations in cAMP response element (CRE) activity were used to assess the functional effects of peptide agonists and 135PAM1 using methods previously described [1], [8], [29]. For these assays, stable clones of SK-N-MC cells (ATCC, Manassas, VA) were selected to express the receptor of interest (RXFP3 or RXFP4) along with a reporter gene linking CRE activity to β-galactosidase expression. CRE activity was stimulated with forskolin in cells expressing RXFP3 or RXFP4 and then inhibition of the CRE activity by agonist was tested with and without 135PAM1. In this reporter assay CRE activity is reflected in β-galactosidase expression. After incubations with compound and/or peptide agonist for 6 hours at 37 C the medium on the cells was discarded. The cells were then lysed and the assay was developed as previously described using the colored substrate chlorophenol red-β-D-galactopyranoside (CPRG) [1].

As described above for Ca^2+^ assays, CRE activity was measured to assess dose related effects of 135PAM1 on peptide agonists at probe concentrations (EC_20_) and to assay 135PAM1-dependent shifting of the agonist dose response curves. In both cases the cells were grown in MEM supplemented with 10% fetal bovine serum, 50 iu/ml penicillin, 50 µg/ml streptomycin, 2 mM L-Glutamine, 1× MEM Non-Essential Amino Acids (Gibco/InVitrogen, Carlsbad, CA), 1 mM Sodium Pyruvate, and 600 µg/ml G418. At confluency, the cells were detached and seeded in 96-well plates at a cell density of 50,000 cells/well for incubation overnight at 37°C, 5% CO_2_. On the following day, the media was discarded and cells were pre-incubated with 135PAM1 at the indicated concentration for 10 min at room temperature. Immediately thereafter, relaxin3 or R3/I5 was added as indicated and incubated for 10 min at room temperature. The cells were then stimulated with forskolin (5 µM, final concentration) and incubated at 37°C, 5% CO_2_ for 6 hours. Following washes with assay buffer, the cells were lysed and β-galactosidase activity was visualized by incubation with CPRG for up to 30 min at room temperature and absorbance was read at 570 nm. Results of 3 or more triplicate functional assays were analyzed together after normalization by non-linear regression (Prism, GraphPad, San Diego, CA) and are reported with confidence intervals in parenthesis.

### Radioligand Receptor Binding Assay

Membrane homogenates were prepared from confluent 15 cm tissue culture dishes of HEK-293 cells expressing G_q_I_5_ (see above for Ca^2+^ functional assays). Cells were removed from the tissue culture dishes with DPBS+5 mM EDTA (pH 7.4), then centrifuged and stored at −80 C. On the day of the assay 14 ml of homogenization buffer (50 mM Tris HCl+5 mM EDTA pH 7.5+Complete Protease Inhibitor cocktail (Roche, South San Francisco, CA) was added to the cell pellet, which was then homogenized for 15 seconds using a Handishear homogenizer (Virtis). The homogenate was spun at 500 xg for 5 minutes at 4 C, then the supernatant fraction was spun at 27000 xg for 30 minutes at 4 C. The resulting membrane pellet was resuspended in 16 ml of homogenization buffer with Compete Protease Inhibitors, briefly homogenized using the Handishear (Virtis), and then kept on wet ice.

Membrane homogenates were used to measure homologous or heterologous binding displacement of [^125^I]R3I5_NH2_. Peptides and tracer were diluted in assay buffer (50 mM Tris HCl+5 mM EDTA pH 7.5) with 0.5% BSA added. Reactions were set up in a total volume of 100 µl. The PAM compound was diluted in 50 C assay buffer as a 5× concentrate and added to the binding reaction promptly to avoid precipitation. [^125^I]R3I5(amide) was added to a net concentration of 14 pM. Binding reactions were incubated for 90 minutes at room temperature, then filtered through GF/C membranes pretreated with polyethyleneimine using a 96 well Brandel apparatus (Brandel, Gaithersberg, MD). The filter plates were dried, then Microscint (Perkin Elmer, Shelton, CT) was added and the plates were counted on a Top Count (Perkin Elmer, Shelton, CT). Data were evaluated using GraphPad Prism 5.01 (GraphPad Software, San Diego, CA). Results of 3 or more triplicate functional assays were analyzed by non-linear regression (GraphPad Prism). Results were analyzed together after normalization and are reported with confidence intervals in parenthesis. Calculation of a cooperativity value between 135PAM and the radioligand peptide was calculated using the allosteric modulator equation based on the ternary complex model in GraphPad Prism.

## Results

### 135PAM1 is a RXFP3 positive allosteric modulator with unique probe dependence

135PAM1's positive allosteric activity was demonstrated in functional assays using probe quantities of the orthosteric agonists and in dose response assays showing shifts in orthosteric agonist pEC_50_ values with increasing quantities of compound. Functional positive allosteric activity was retained following substitution of the entire A-chain of relaxin-3 (e.g. R3/I5_NH2_), however c-terminal amidation of the RXFP3 orthosteric agonist peptide was required for PAM activity of 135PAM1.

**Figure 2:**
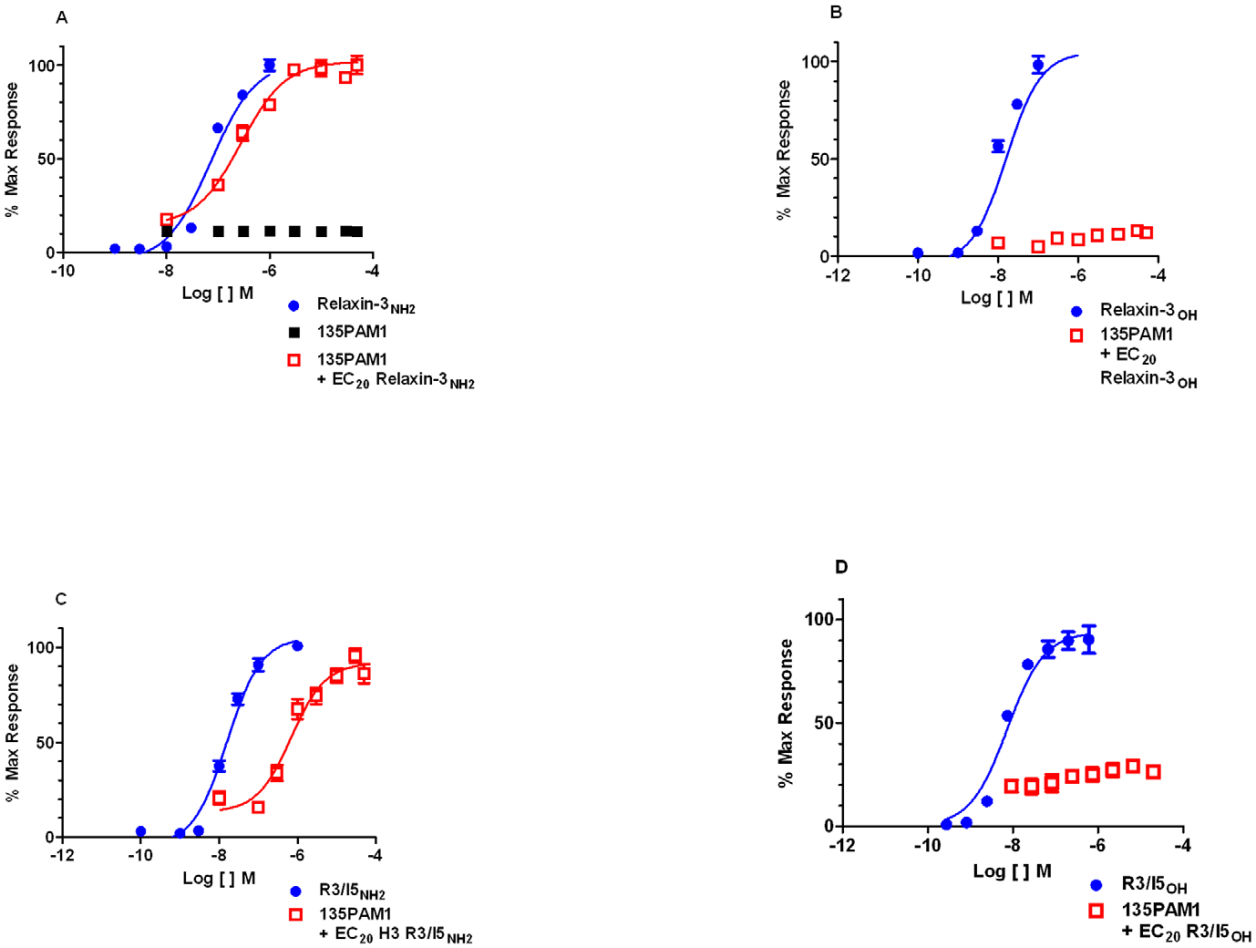
135PAM1 increases the intracellular Ca^2+^ response to amidated, but not free acid RXFP3 agonists in cells coexpressing RXFP3 and G_qI5_. Intracellular Ca^2+^ responses by HEK-293 cells coexpressing RXFP3 and G_qI5_ were measured in response to escalating concentrations of 135PAM1 using probe (EC_20_) concentrations of Relaxin-3_NH2_ (A), Relaxin-3_OH_ (B), R3/I5_NH2_ (C), or R3/I5_OH_ (D).

First, ascending doses of 135PAM1 were shown to increase calcium mobilization elicited by a probe concentration of Relaxin-3 (amide or free-acid) or R3I5 (amide or free-acid) (Figure 2). HEK293 cells co-expressing RXFP3 and G_qi5_ were incubated with increasing concentrations of 135PAM1, followed by addition of relaxin-3 or R3/I5 at the EC_20_ (the EC_20_ was determined using a separate plate on the day of the assay). 135PAM1 potentiated the RXFP3-mediated calcium response to amidated relaxin-3 and R3/I5 (Fig. 2 A and C), but not to free acid analogs of either peptide (Fig. 2 B and D). As shown in Fig. 2A, 135PAM1 lacked agonist activity in the absence of a RXFP3 orthosteric agonist. 135PAM1 was more potent at RXFP3 in the presence of relaxin-3_NH2_ (pEC_50_ 6.55 [6.46 to 6.64]) than R3/I5_NH2_ (pEC_50_ 6.07 [5.94 to 6.20]). The E_Max_ of 135PAM1 in the presence of Relaxin-3_NH2_ at its EC_20_ was significantly less than the E_Max_ of the peptide (P<0.01; Relaxin-3_NH2_ E_Max_ = 25811 AU [24571–27051], 135PAM1 E_Max_ with probe = 22362 AU [21642–25221]). Similarly, the E_Max_ of 135PAM1 with R3/I5_NH2_ at its EC_20_ was significantly less than the E_Max_ of R3/I5_NH2_ (P<0.03; R3/I5_NH2_ E_Max_ = 23086 AU [22257–23914], 135PAM1 E_Max_ with probe = 21194 AU [19389–22999].

**Figure 3:**
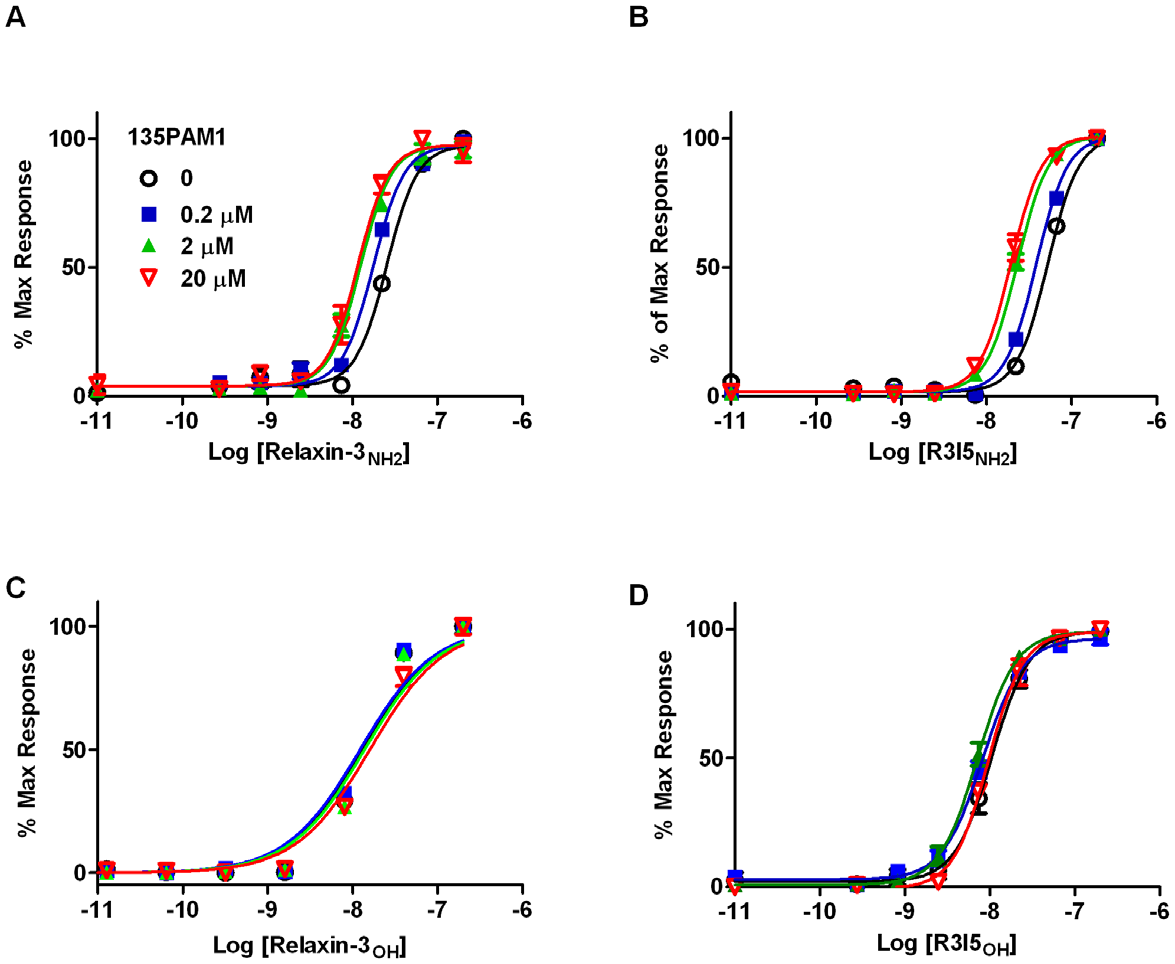
135PAM1 shifts the concentration response curves of Relaxin-3_NH2_ and R3/I5_NH2_. HEK-293 cells coexpressing RXFP3 and G_qI5_ were incubated with fixed concentrations of 135PAM1 (0, 0.2, 2 and 20 µM) 10 min before the addition of increasing concentrations of Relaxin-3_NH2_ (A), R3I5_NH2_ (B), Relaxin-3_OH_ (C) or R3I5_OH_ (D).

135PAM1 also shifted the relaxin-3_NH2_ and R3I5_NH2_ concentration-response curves in a limiting manner that is indicative of allosteric activity (Figure 3). For these experiments, cells from the same clone of HEK-293 cells expressing RXFP3 and G_qI5_ were preincubated with fixed concentrations of 135PAM1 (0, 0.2, 2.0, 20 µM) and then stimulated with increasing concentrations of relaxin-3_NH2_ or R3/I5_NH2_. 135PAM1 elicited a similar dose-dependent leftward shift in the relaxin-3_NH2_ (1.4–2.3 fold) and R3/I5_NH2_ (1.3–2.6 fold) concentration-response curves (Fig. 3 A and B), but did not significantly shift free agonist peptide dose response curves (Fig. 3C and 3D). An allosteric ternary complex model estimated the RXFP3 p*K*_b_ value of 135PAM1 to be 6.45 (6.16 to 6.74) using the relaxin-3_NH2_ probe or 6.02 (5.87 to 6.18) for the R3/I5_NH2_ probe.

RXFP3 is a G_i/o_ linked GPCR [31], [32], therefore a cAMP response element (CRE) reporter assay was used to confirm the positive allosteric modulator activity of 135PAM1 in bioassays avoiding use of a chimeric G-protein. Results of these studies were consistent with those obtained above using G_qI5_.

**Figure 4:**
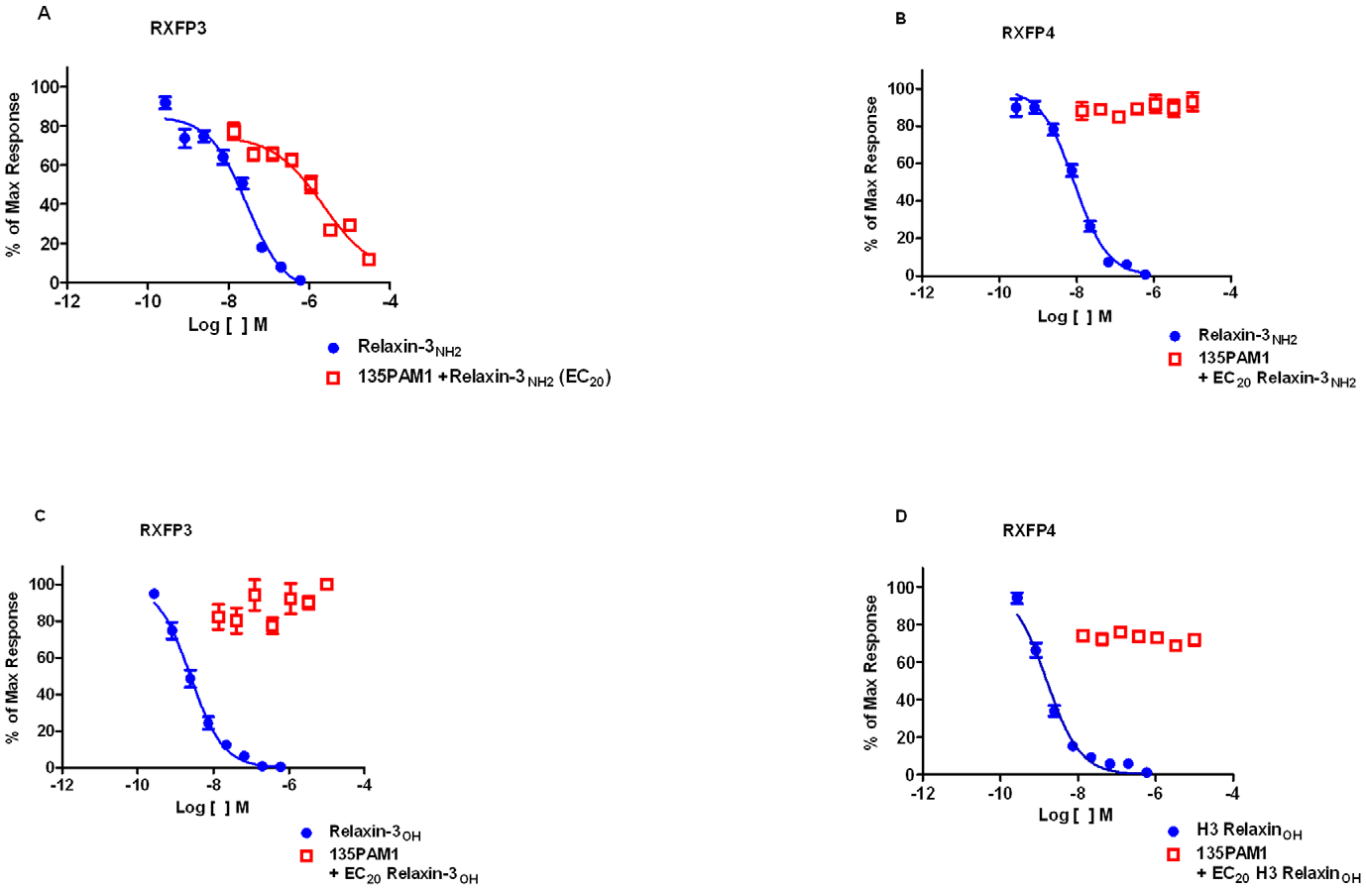
135PAM1 dose-dependently augments the inhibition of CRE activity of probe (EC_20_) concentrations of Relaxin-3_NH2_ in cells expressing RXFP3, but not RXFP4. As RXFP3 and RXFP4 are G_i/o_-linked receptors, experiments analogous to Fig. 2 were performed without the use of a chimeric G protein, comparing cAMP response element (CRE) activity in cells expressing RXFP3 (panels A and C) to closely related receptor RXFP4 (panels B and D). RXFP3 or RXFP4 receptors were expressed in SK-N-MC cells co-expressing a CRE construct linked to β-galactosidase expression. Forskolin (5 µM) was used to stimulate CRE activity and ascending doses of 135PAM1 were added to probe (EC_20_) concentrations of Relaxin-3_NH2_ (A and B) or Relaxin-3_OH_ (C and D).

Similar to the Ca^2+^ mobilization assays described above, CRE activity was first evaluated using escalating concentrations of 135PAM1 and probe concentrations of relaxin-3. When added to 10 nM Relaxin-3_NH2_, 135PAM1 inhibited the CRE response to 5 µM forskolin with a pIC_50_ of 6.12 (5.98 to 6.27), but had no effect on the CRE response to Relaxin-3_OH_ (see Fig. 4 A and C). The E_Max_ observed for inhibition of the CRE response to 5 µM forskolin by 135PAM1 at 30 µM was less than the E_Max_ of Relaxin-3_NH2_, however the dose response curve had not yet reached its asymptote and testing of 135PAM1 at a higher concentration was not possible due to solubility limitations (Fig. 4A). While calculation of the 135PAM1 E_Max_ is impractical in this case, the results are similar to those observed above for Ca^2+^ measurements in HEK293 cells co-expressing RXFP3 and G_qi5_.

**Figure 5:**
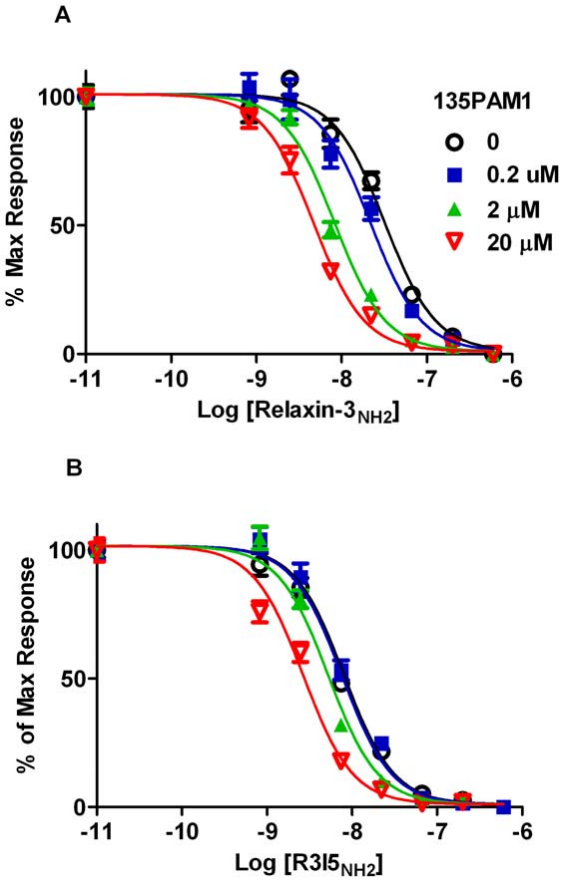
Shifting of amidated RXFP3 agonist concentration response curves by 135PAM1 in cells lacking chimeric G proteins. SK-N-MC cells coexpressing RXFP3 and a reporter construct linking CRE activity to β-galactosidase were incubated with fixed concentrations of 135PAM1 (0, 0.2, 2 and 20 µM) and increasing concentrations of Relaxin-3_NH2_ (A) or R3I5_NH2_ (B).

135PAM1 also shifted Relaxin-3_NH2_ (Fig. 5A) and R3/I5_NH2_ (Fig. 5B) inhibition of forskolin-stimulated CRE responses to the left, increasing the efficacy of these peptide agonists. An allosteric ternary complex model estimated the RXFP3 p*K*_b_ value of 135PAM1 to be 5.59 (5.37 to 5.80) when shifting the relaxin-3_NH2_ curve or 5.05 (4.70 to 5.40) when shifting the R3/I5_NH2_ curve.

### 135PAM1 has no affinity at the orthosteric binding site of the RXFP3 receptor

**Figure 6:**
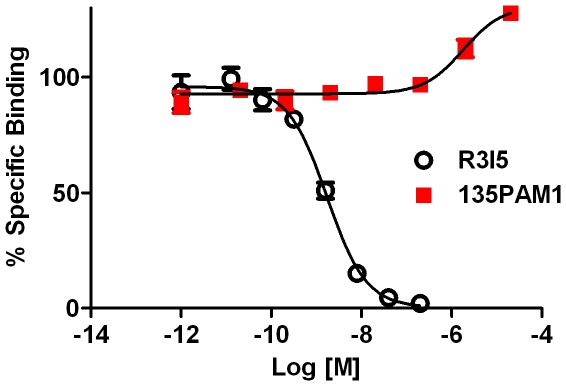
135PAM1 lacks affinity at the orthosteric binding site of RXFP3 receptor. 135PAM1 did not displace [125I] R3/I5_NH2_ at concentrations of up to 20 µM, but instead increased total binding. R3/I5_NH2_ displaced the tracer with a pIC_50_ of 8.76 (8.91 to 8.61).

We assessed the ability of 135PAM1 to compete for binding with the orthosteric radioligand, [^125^I] R3I5 (amide), at the orthosteric site using membranes prepared from RXFP3-HEK293-G_qi5_ cells (Fig. 6). 135PAM1 failed to displace 14 pM [^125^I] R3I5 (amide) at concentrations of up to 20 µM (Fig. 6). In contrast, the orthosteric agonist, R3I5 (amide), displaced [^125^I] R3I5 (amide) with a pIC_50_ of 8.76 (8.61 to 8.91) (Fig. 6). At higher concentrations of 135PAM1 an increase in binding was observed, consistent with positive allosterism [33]. Further calculation of these data for 135PAM1 showed positive cooperativity with an α of 1.4 [23].

### 135PAM1 is selective for RXFP3

Receptor selectivity of 135PAM1 was explored using RXFP4, which has the highest known sequence identity (43%) compared to RXFP3 [29]. Relaxin-3 and R3/I5 are full RXFP4 agonists. While Relaxin-3 in either the free acid or c-terminally amidated forms inhibited the CRE response to 5 µM forskolin in cells expressing RXFP4, 135PAM1 (at up to 20 µM) had no effect on either analog of relaxin-3 at EC_20_ concentrations (Fig. 4B and 4D).

Ancillary pharmacology was further evaluated using broad profiling screen at CEREP and in house, evaluating a total of 60 GPCRs, ion channels and transporters in radioligand binding assays, resulted in only four significant activities (dopamine transporter 83% at 10 µM, norepinephrine transporter 59% at 10 µM, serotonin 5-HT1A 74% at 10 µM and sodium channel site 2 83% inhibition at 10 µM). In addition, the compound was tested against OX2R and NPSR in an in vitro functional assay (FLIPR). In these 2 assays, when incubated in the presence of EC_20_ concentrations of either orexin A or NPS, 135PAM1 did not show any PAM activity at concentrations up to 20 µM.

## Discussion

Neuropeptides have long been recognized to have therapeutic potential. Successful uses of peptides have been primarily in peripheral applications, for example, in the tachykinin, somatostatin, angiotensin, calcitonin gene-related peptide (CGRP), orexin, or glucagon-like peptide1 fields [34]. Manipulation of native peptide agonists to take advantage of key domains has yielded receptor antagonists such as α-helical CRF(9–41) [35] and R3(Δ23–27)R/I5 [21]. R3/I5, the RXFP3/4 agonist used in these studies, was similarly constructed to retain the domain needed for RXFP3/4 activity while eliminating the domain needed for RXFP1/2 activity [22]. Given the complex nature of receptor–ligand interactions at the orthosteric site of GPCRs, it is not surprising that the number of such ligands, especially brain penetrant agonists, is very limited. Many synthetic neuropeptide analogs exist, but most of them are peptides, with inherent limitations (i.e. bioavailability, stability, brain penetration). In the last decade, allosteric modulation of GPCRs has offered great opportunities for receptors for which the chemical space is limited. Negative allosteric modulators of CRF, CGRP, GLP1 or ghrelin receptors have been discovered [36]. When it comes to agonists, almost no neuropeptide receptor PAMs are described. L-692,429, a ghrelin receptor PAM, is one of the very few peptide receptor PAMs to be described [37]. Additionally, low molecular weight ago-allosteric compounds acting on GLP1 receptors exist [38].

Recent data point to the therapeutic potential of an RXFP3 agonist or positive modulator [39], however, selective, systemically active, nonpeptide relaxin-3 receptor ligands have not yet been discovered [39]. Here, we describe the in vitro characterization of the first RXFP3 receptor PAM, 135PAM1. 135PAM1 was discovered in an antagonist screen of more than 100,000 compounds when negative inhibition of R3/I5_NH2_ activity on RXFP3 was noticed. Approximately 0.1% of the compounds tested in the screen displayed the negative inhibition and 135PAM1 proved to be a selective RXFP3 positive allosteric modulator. This low-molecular-weight ligand moderately potentiates the functional responses of amidated Relaxin-3 or amidated R3/I5 in calcium mobilization and cAMP assays in cell lines expressing the cloned human RXFP3 receptor. 135PAM1 does not compete for binding with orthosteric radioligand [^125^I]R3/I5_NH2_ but instead increases its binding and shows positive cooperativity, consistent with PAM activity. However, 135PAM1 lacks similar activities when using the free acid form of relaxin-3 (the native form) or R3/I5 instead of the amidated form, indicating that the compound is subject to probe dependency related to the state of the probe's c-terminus. This compound demonstrates a site for allosteric modulation of RXFP3 and shows the importance of following such compounds using a free acid probe.

Positive allosteric modulation of RXFP3 by 135PAM1 has been shown using a chimeric G_qI5_ protein to shift signaling to intracellular Ca^2+^ and in assays measuring native G_i/o_ signaling. When given with probe concentrations of Relaxin-3_NH2_ or R3/I5_NH2_, 135PAM1 has an Emax slightly lower than either peptide (fig. 2A, 2C and 4A). The PAM compound shifts the concentration response of either amidated agonist peptide in a dose related fashion (fig. 3 and 5). The apparent pK_b_ for the allosteric site is higher when using the G_qi5_-linked functional assay (pK_b_ = 6.45 for the relaxin-3_NH2_ shift; Fig. 3A) than in the native G_i/o_-linked assay (pK_b_ = 5.6 for the relaxin-3_NH2_shift; Fig. 5A). The pK_b_ is high enough in the G_qi5_-linked functional assay to show saturation of the PAM effect (Fig. 3A/B), however the limited solubility of 135PAM1 makes it impractical to demonstrate saturability of that effect in the native G_i/o_-linked assay (Fig. 5A).

In addition to the Ca^2+^ mobilization assay, we utilized a CRE reporter assay to evaluate the pharmacology of the RXFP3 positive allosteric modulator, 135PAM1. RXFP3 is functionally coupled to the inhibition of cAMP through the inhibition of adenylate cyclase when coupling to G_i/o_ proteins. We observed that increasing concentrations of both Relaxin-3_NH2_ and R3I5_NH2_ inhibit CRE activation and that 135PAM1 potentiates this effect. However, CRE can be transactivated by multiple signaling pathways, including cAMP and ERK [40]. Van der Westhuizen et al demonstrated that Relaxin-3 induces ERK1/2 phosphorylation via activation of Gi/o proteins [41]. Taking this into consideration, Relaxin-3 and related peptides could induce the activation of the CRE response in our assay via ERK1/2. However, since the van der Westhuizen's group reported maximum P-ERK1/2 levels for just minutes following Relaxin-3 administration and our CRE reporter assay incubates for 6 hours at 37 C, we speculate any effect of ERK signaling observed was minimal.

The lack of affinity of 135PAM1 for the orthosteric site of RXFP3 and the lack of effect of 135PAM1 on baseline cAMP or calcium suggests 135PAM1 is a positive allosteric modulator of RXFP3 without intrinsic activity. Also, receptor selectivity of 135PAM1 at RXFP3 has been demonstrated by comparison with cells expressing RXFP4, a GPCR with high homology to RXFP3 [29]. The selectivity of the effect of 135PAM1 at RXFP3 was evidenced by its lack of effect on Relaxin-3 or R3/I5 (amidated or free acid) responses in cells stably expressing RXFP4 receptors (Fig. 4B and 4D). In addition to RXFP4, 135PAM1 was tested for activity against 60 other receptors and channels, with no remarkable activity reported.

Initial studies demonstrated that the amidated and free-acid forms of Relaxin-3 were equipotent [42]. However, recent results both here and elsewhere (Ross Bathgate, personal communication) have shown amidated RXFP3 agonists (Relaxin-3 and R3/I5) to be roughly 10 fold less efficacious (lower pEC_50_) than their free acid analogs. Early work with native free acids of these insulin-like peptides used recombinant material made in eukaryotic cells, which was purified to homogeneity [1], [22]. Insulin-like peptides have since become synthetically tractable [43] and such methods have allowed for modifications such as c-terminal amidation of the peptide chains. 135PAM1 was discovered using amidated peptide, as amidation of peptides tends to increase solubility, increase stability and prior data indicated amidation of RXFP3 agonists did not alter potency [42]. All materials described here were made synthetically and were checked by LCMS. Molecular masses were correct and consistent with both chains of both peptides being free acids or amidated, as indicated. The reason for the observed differential potencies of c-terminal amides versus acid peptides remains unclear.

While allosteric modulators with probe dependency have been described in the literature [27], [44], [45], 135PAM1 is a unique example. Allosteric activity is retained while substituting the entire A-chain of Relaxin-3_NH2_ with the A-chain of INSL-5_NH2_ (yielding R3/I5_NH2_), yet 135PAM1 is inactive when the native free acid forms of these peptides are used as probes. Perhaps the most cited examples of probe dependent allosteric modulators are for M4 acetylcholine receptors [45], [46] and the CCR5 chemokine receptor [47]. In the case of the M4 receptor, LY2033298 shows greater cooperativity with oxotremorine than with acetylcholine or xanomeline. Oxotremorine, acetylcholine and xanomeline are small molecules with substantial chemical differences. In the chemokine receptor example, 873140 shows probe dependent allosteric antagonism at CCR5 using a site common to four other known inhibitors [47]. However, the various endogenous ligands used as probes for chemokine receptors such as CCR5 are peptides with substantial sequence variability [48]. In either case the probe agonists for the receptor in question are far more variable than the differential c-terminal amidation shown here.

Probe selective allosteric modulators of the GLP-1 receptor are in some ways comparable to the probe dependence described here. Structurally similar flavonoids show positive cooperativity with GLP-1 fragments and exendin-4, but not oxyntomodulin [49]. A hydroxyl group is crucial to positive cooperativity, but this differs from the RXFP3 case in that the needed hydroxyl group is on the allosteric modulator, as opposed to the probe.

Here, we have shown the free acid and amide forms of Relaxin-3 display different potencies at the RXFP3 receptor and described 135PAM1, a novel positive allosteric modulator of RXFP3. This RXFP3 PAM is selective to RXFP3 over RXFP4 and a panel of other receptors, shows activity in assays measuring G_qI5_-linked changes in intracellular Ca^2+^ or native G_i/o_ linked cAMP, and shows probe selectivity. 135PAM1 shows a probe selectivity dependent on the c-terminal amidation state of the peptide agonists, unfortunately favoring c-terminal amides which have not been shown to occur in nature. 135PAM1 is the first publicly disclosed positive allosteric modulator of RXFP3. Since 135PAM1 lacks activity when used with the native, free acid form of relaxin3 and has severe solubility limitations, the compound has very limited utility. 135PAM1 would lack in vivo activity when given alone, but could be used to potentiate effects of exogenous Relaxin-3_NH2_ or R3/I5_NH2_. This example demonstrates the subtlety of differences in probe agonists which can lead to probe dependence.
